# A plasmatic score using a miRNA signature and CXCL-10 for accurate prediction and diagnosis of liver allograft rejection

**DOI:** 10.3389/fimmu.2023.1196882

**Published:** 2023-05-30

**Authors:** Olga Millán, Pablo Ruiz, Judit Julian, Ana Lizana, Yiliam Fundora, Gonzalo Crespo, Jordi Colmenero, Miquel Navasa, Mercè Brunet

**Affiliations:** ^1^ Biomedical Research Center in Hepatic and Digestive Diseases (CIBEREHD), Instituto de Salud Carlos III (ISCII), Madrid, Spain; ^2^ Pharmacology and Toxicology, Biochemistry and Molecular Genetics, Biomedical Diagnostic Center (CDB), Hospital Clinic of Barcelona, Instituto de Investigaciones Biomédicas August Pi i Sunyer (IDIBAPS), University of Barcelona, Barcelona, Spain; ^3^ Liver Unit, Hospital Clinic of Barcelona, Instituto de Investigaciones Biomédicas August Pi i Sunyer (IDIBAPS), University of Barcelona, Barcelona, Spain; ^4^ Biochemistry and Molecular Genetics, Biomedical Diagnostic Center, Hospital Clínic of Barcelona, Barcelona, Spain; ^5^ Department of General and Digestive Surgery, Hospital Clínic Barcelona, Instituto de Investigaciones Biomédicas August Pi i Sunyer (IDIBAPS), University of Barcelona, Barcelona, Spain

**Keywords:** noninvasive biomarkers, score, miRNAs, CXCL-10, liver transplant (LT), rejection, prediction, diagnosis

## Abstract

**Introduction:**

The use of noninvasive biomarkers may avoid the need for liver biopsy (LB) and could guide immunosuppression adjustment in liver transplantation (LT). The aims of this study were: to confirm the predictive and diagnostic capacity of plasmatic expression of miR-155-5p, miR-181a-5p, miR-122-5p and CXCL-10 for assessing T-cell mediated rejection (TCMR) risk; to develop a score based on a panel of noninvasive biomarkers to predict graft rejection risk and to validate this score in a separate cohort.

**Methods:**

A prospective, observational study was conducted with a cohort of 79 patients followed during the first year after LT. Plasma samples were collected at predetermined time points for the analysis of miRNAs and the CXCL-10. Patients with LFTs abnormalities were submitted to a LB to rule out rejection, assessing previous and concurrent expression of the biomarkers to evaluate their predictive and diagnostic ability. Information from 86 patients included in a previous study was collected and used as a validation cohort.

**Results:**

Twenty-four rejection episodes were diagnosed in 22 patients. Plasmatic CXCL-10 concentration and the expression of the three miRNAs were significantly elevated prior to and at the moment of the diagnosis of rejection. We developed a logistic model for rejection prediction and diagnosis, which included CXCL-10, miR-155-5p and miR-181a-5p. The area under the ROC curve (AUROC) for rejection prediction was 0.975 (79.6% sensitivity, 99.1% specificity, 90,7% PPV; 97.7% NPV; 97.1% correctly classified) and 0.99 for diagnosis (87.5% sensitivity, 99.5% specificity, 91.3% PPV; 99.3% NPV; 98.9% correctly classified). In the validation cohort (n=86; 14 rejections), the same cut-off points were used obtaining AUROCs for rejection prediction and diagnosis of 0.89 and 0.92 respectively. In patients with graft dysfunction in both cohorts the score could discriminate those with rejection regarding other causes with an AUROC of 0.98 (97.3% sensitivity, 94.1%specificity).

**Conclusion:**

These results suggest that the clinical implementation of the monitoring of this noninvasive plasmatic score may allow the prediction and diagnosis of rejection and identify patients with graft dysfunction due to rejection, helping with a more efficient guide for immunosuppressive therapy adjustment. This finding warrants the development of prospective biomarker-guided clinical trials.

## 1 Introduction

Several promising biomarkers have been identified for detecting the degree of alloreactivity in transplantation patients, for determining personal response to treatment and individual drug doses, and diagnosing graft dysfunction (GD) and injury.

The Scientific Community in Solid Organ Transplantation currently considers several biomarkers such as cytokines, chemokines, T and B-cell immunophenotypes, and gene expression, among other molecular biomarkers, to have potential as diagnostic and prognostic biomarkers of graft evolution (1–3). Blood genomic assays measuring donor-derived cell-free DNA (dd-cfDNA), microRNA (miRNA) and mRNA have shown promising results in predicting rejection (4–7).

In kidney transplantation some formulas and scores for noninvasive accurate diagnosis and prediction of rejection have been proposed. All these scores have been developed based on urine samples and included some molecular biomarkers such as chemokines; donor-specific antibodies (DSAs); dd-cfDNA, and mRNA levels of genes involved in acute rejection and graft injury (8–11). These studies illustrate the great potential of more mathematical approaches to calculate rejection probability instead of relying on “graft functional impairment” alone and could also be very useful in guiding immunosuppressive management.

To the best of our knowledge, no score has yet been described that includes biomarkers for the prediction and diagnosis of rejection, and GD in liver transplantation (LT). In LT, acute rejection remains the leading cause of graft dysfunction during the first months after transplantation, affecting 21%-27% of patients (12, 13). Patients who have clinical evidence of allograft dysfunction must undergo liver biopsy (LB) to confirm the diagnosis of acute rejection, despite increased concerns regarding interobserver variability between histological evaluations (14). The liver is an immunologically complex organ involved in the synthesis of acute-phase proteins, cytokines and chemokines. Hepatic inflammatory mechanism may initiate and mediate immune responses with an impact on the long-term allograft outcome (15). Noninvasive biomarkers that reflect alloimmune activation early or maintain alloreactivity may provide patient risk stratification and personalized immunosuppression (IS)(early posttransplantation, minimization, and long-maintenance IS).

miRNAs and chemokines have been the focus of interest as noninvasive biomarkers for graft outcome. miRNAs play a regulatory role in mediating gene expression at the posttranscriptional level and are involved in numerous biological processes such as cell differentiation, proliferation and apoptosis, processes that in turn are involved in acute allograft rejection and injury (16–18). Consequently, physiological and pathological changes can induce alterations in circulating miRNA. Several studies have been performed on different signatures of miRNAs as diagnostic biomarkers of liver diseases in patients with hepatitis B and hepatitis C virus infections, nonalcoholic steatohepatitis, nonalcoholic fatty liver disease and hepatocellular carcinoma (19).

Previous studies have shown the potential role of miRNA analysis as a noninvasive prognostic and diagnostic biomarker for rejection. Farid et al. (6) demonstrated that circulating hepatocyte-derived miR-122, miR-148a, and miR-194 correlate with hepatic injury and acute rejection. Shaked et al. (7) by using the framework of the Immune Tolerance Network Immunosuppression Withdrawal (ITN030ST) and Clinical Trials in Organ Transplantation (CTOT-03) studies, identified two miRNAs (miR-483-3p and miR-885-5p) in plasma that, when combined in a signature, could be used to diagnose and predict liver rejection with high accuracy. This miRNA signature is predictive of the risk of rejection in LT patients during IS withdrawal. Several groups have demonstrated that miR-155-5p is overexpressed in liver tissue, serum and circulating inflammatory cells during liver injury (20, 21). In an early observational study conducted in a cohort of 145 *de novo* adult LT recipients, our group reported that pretransplantation, plasmatic miR-155-5p and miR-181a-5p expression may be useful for stratifying low-immunologic-risk patients and in the early posttransplantation period (from the 1^st^ week to 1^st^ month), before transaminase-level modification, a significantly increased of miR-181a-5p, miR-155-5p, and miR-122-5p expression was observed in patients with rejection (22). Moreover, cut-off values for the risk of T-cell mediated rejection (TCMR) and subclinical rejection (SCR) for plasmatic expression of miR-155-5p, miR-122-5p and miR-181a-5p were established (22). In addition, the results from this cohort demonstrate that miRNA expression levels in plasma can be used to differentiate TCMR from other causes of GD early after LT (23). miR-122-5p is the most abundant liver-derived miRNA, constituting 70% of the total miRNA in the liver (24), and thus, a significant increase in this miRNA could be associated with hepatocyte damage, toxicity or viral infection (25). Schumuck et al. (26) also demonstrated that the levels of miR-122-5p are significantly higher in the bile of liver recipients who develop AR within the first 6 months after transplantation and during an AR episode.

Chemokines can also contribute to the risk of rejection assessment through their important role in the recruitment of lymphocytes to sites of injury and inflammation which occurs in rejection episodes. Several kidney transplantation studies have demonstrated the potential of urine and plasmatic CXCL-10 measurement as predictive and diagnostic biomarkers of TCMR and antibody mediated rejection (ABMR) (27–32). In LT, studies assessing chemokines as biomarkers of risk of rejection are scarce. The results from Raschzok et al. (33) suggest the potential of serum protein levels of CD44 and CXCL-9 chemokine monitoring as predictive biomarkers of allograft rejection after LT.

As previously mentioned, both kinds of biomarkers can help in the early identification of patients at high risk of acute rejection and enable personalized therapy, thus improving treatment efficacy and safety (9, 34). Moreover, our previous results showed their potential use in predicting graft evolution and to identifying modifiable risk variables to improve immunosuppressive treatment in liver and kidney transplant recipients (22, 23, 29, 30).

The aims of this study were: 1) to confirm the predictive and diagnostic capacity of plasmatic expression of miR-155-5p, miR-181a-5p, miR-122-5p and CXCL-10 for the assessment of the risk of TCMR in LT recipients; 2) to develop a score based on a panel of noninvasive biomarkers to predict the risk of graft rejection and GD; and 3) to validate this score in a previously selected cohort.

## 2 Patient and methods

An observational, prospective study was conducted with a cohort of patients who subsequently received transplantation at a single center (Hospital Clínic Barcelona). Individuals requiring for double liver-kidney transplantation were excluded, as were those patients who died during the first week of LT. From September 2020 to March 2022, a total of 79 patients were included. All patients were followed during the first year after LT. Clinical, demographic and laboratory data were collected and are summarized in **Table 1**. The majority of patients were males (75%) with a median age of 58 years. The main reasons for LT were alcohol-related cirrhosis (33%) and hepatitis C virus infection (16.5%). Hepatocellular carcinoma was the indication for LT in 30.3% of patients. Most individuals received grafts from donors after brain death (73.4%), and the median donor age was 60 years. The median cold ischemia time (CIT) was 392 minutes. All patients signed informed consent approved by the IRB (number).

**Table 1:** Characteristics of the study cohort.

	TOTAL (79)	Rejectors (22)	Non-rejectors (57)	p-value
Sex (Male)	59 (75%)	15 (68%)	44 (77%)	0.41
Age (Years)	58 (50-64)	56.5 (48-64)	59 (52-63)	0.77
Primary disease				
Alcohol	26 (33%)	8 (36.3%)	18 (31.6%)	0.68
HCV	13 (16.5%)	2 (9%)	11 (19.3%)	0.32
HBV	6 (7.6%)	2 (9%)	4 (7%)	0.39
Autoimmune	5 (6.3%)	2 (9%)	3 (5,3%)	0.69
Cholestasic	7 (8.9%)	2 (9%)	5 (8.8%)	0.63
Cryptogenic	3 (3.8%)	1 (4.6%)	2 (3.5%)	0.63
MAFLD	8 (10.1%)	1 (4.6%)	7 (12.3%)	0.43
Others	10 (12.7%)	4 (18.2%)	6 (10.5%)	0.35
HCC	24 (30.3%)	11 (50%)	13 (22.8%)	0.018
Donor type (DBD)	58 (73.4%)	15 (68.2%)	43 (75.4%)	0.51
Donor age (Years)	60 (51-70)	58 (44-72)	62 (54-69)	0.47
CIT (min)	392 (346-455)	397 (360-480)	390 (341-455)	0.56
IS protocol Double Triple	23 (29.1%) 56 (70.9%)	14 (24.6%) 43 (75.4%)	9 (40.9%) 13 (59.1%)	0.17
ABS	14 (17.7%)	2 (9.1%)	12 (21%)	0.18
CMV infection	17 (21.5%)	1 (4.6%)	16 (28.1%)	0.03

Characteristics of the study cohort. Variables are displayed as medians and interquartile ranges. Count variables are displayed raw and intragroup relative frequency. HCV, hepatitis C virus; HBV, hepatitis B virus; MAFLD, metabolic associated fatty liver disease; HCC, hepatocellular carcinoma; DBD, donor after brain death; CIT, cold ischemia time; IS protocol, immunosuppression protocol; Double, TAC + Prednisone; Triple, TAC + MMF + Prednisone; ABS, anastomotic biliary strictures; CMV, cytomegalovirus. A p value < 0.05 was considered statistically significant.

### 2.1 Immunosuppression

IS regimens were defined according to the pre-LT liver status. Patients with a Child-Pugh A classification were given a double therapy with corticosteroids and TAC starting within 24 hours after LT with target trough levels of 8-10 ng/ml. Those with Child-Pugh B-C, patients transplanted due to acute liver failure or retransplantation received induction with a single basiliximab dose (20mg), and triple therapy with corticosteroids, mycophenolate mofetil (MMF) 2000 mg daily and TAC starting at day 5 after LT with target trough levels of 5-8 ng/ml. In all individuals the corticosteroid dose was tapered to be withdrawn at month 6 after surgery. The MMF dose was reduced at month 1 to 1500 mg daily. Everolimus (EVR) could be started beyond three weeks after LT in those individuals in which TAC or MMF had to be reduced or withdrawn due to adverse effects.

### 2.2 Follow up, graft dysfunction (GD) and liver biopsies

All patients were followed up by transplant hepatologists according to standardized throughout the first year after LT. Study visits for liver function testing, pharmacokinetic monitoring and plasma collection were performed at weeks 1 and 2 and months 1, 2, 3, 6, 9 and 12. An additional plasma sample was taken before LT for the miRNA expression and chemokine analysis. GD was diagnosed if aspartate aminotransferase (AST), alanine aminotransferase (ALT), or bilirubin serum levels were 2-fold higher than the upper limit of normal during the follow up routine laboratory surveillance, or if these parameters did not show decrease throughout the first two weeks after LT. An abdominal ultrasound examination ruling out vascular or biliary complications that might explain such biochemical abnormalities was mandatory for the GD diagnosis. Those patients with GD underwent an LB to rule out rejection. All biopsies were reviewed by an expert pathologist and the rejection diagnosis and severity were defined using the Banff Working Group criteria (35). Other causes of GD aside from rejection as a result of the biopsy were also recorded. Those patients with suspicion of anastomotic biliar stricture (ABS) were submitted either to magnetic resonance cholangiopancreatography (MRCP) or endoscopic retrograde cholangiopancreatography (ERCP) to confirm the diagnosis. All patients were monitored weekly for CMV viral load in plasma for the first 2 months and at least monthly for 6 months after LT. CMV infection was defined as CMV DNA>1000 copies/ml, and was treated with oral valganciclovir for 14 days or until the viral load was undetectable. CMV disease was treated with iv ganciclovir.

### 2.3 Validation cohort

A validation cohort from a previous study was selected (22) and consisted of patients who were prospectively followed up with plasma collection during the first year after LT (**Table 2**). Only patients for whom chemokine and miRNA analyses were available were included. Patients who were diagnosed with subclinical rejection were excluded because the cohort recruited in the present study was not submitted to a per-protocol LB, thus excluding the possibility of finding this entity.

**Table 2:** Characteristics of study and validation cohorts.

	Total (n=165)	Validation Cohort (n=86)	Study Cohort (n=79)	P value
Sex (Male)	125 (75.2%)	65 (75.6%)	59 (74.7%)	0.8
Age (median/IQR)	58 (51-63)	56 (51-62)	58 (50-64)	0.27
Primary disease				
Alcohol	47 (28.5%)	21 (24.4%)	26(33%)	0.21
HCV	43 (26.1%)	29 (33.7%)	14(17.7%)	0.02
HBV	11 (6.7%)	5 (5.8%)	6(7.6%)	0.55
Autoimmune	8 (4.9%)	3 (3.5%)	5(6.3%)	0.71
Cholestasic	16 (9.7%)	9 (10.5%)	7(8.9%)	0.79
Cryptogenic	6 (3.6%)	4 (3.5%)	3(3.8%)	0.55
MAFLD	16 (9.7%)	8 (9.3%)	8(10.1%)	0.83
Others	18 (10.9%)	8 (9.3%)	10(12.7%)	0.47
HCC	60 (36.4%)	36 (41.9%)	24 (30.4%)	0.14
DCD	34 (20.9%)	13(15.5%)	21(26.6%)	0.08
CIT (minutes)	420 (360-490)	435(374-520)	393(346-455)	0.004
IS protocol				
Double	45 (27.3%)	22 (25.6%)	23 (29.1%)	0.58
Triple	120 (72.7%)	64 (74.4%)	56 (70.9%)	
Rejection	36 (21.8%)	14 (16.3%)	22 (27.8%)	0.06

Characteristics of the total cohort (n=165), the study cohort (n=79) and the validation cohort (n=86). HCV, hepatitis C virus; HBV, hepatitis B virus; MAFLD, metabolic associated fatty liver disease; HCC, hepatocellular carcinoma; DCD, donor after cardiac death; CIT, cold ischemia time; IS protocol, immunosuppression protocol; Double, TAC + Prednisone; Triple, TAC + MMF + Prednisone. A p value < 0.05 was considered statistically significant.

### 2.4 Pharmacokinetic monitoring

Trough concentrations at the 1^st^ week, on the 15^th^ day, and at the 1^st^, 2^nd^, 3^rd^, 6^th^, 9^th^ and 12^th^ months after LT were analyzed. Whole-blood TAC concentrations were determined by Tacrolimus-CMIA-Architect from Abbot (Wiesbaden, Germany) following the manufacturer’s instructions, and whole-blood EVR concentrations were determined by liquid chromatography/tandem mass spectrometry (HPLC/MS/MS). Fresh samples, without having been previously frozen, were analyzed daily. LGC Standard Proficiency Testing was ensured by the participation of our laboratory in the United Kingdom External Analytical Quality Assessment Service.

### 2.5 Plasmatic chemokine measurements

For the analysis of plasmatic CXCL-10 at the time of the clinical visits and biopsies whole blood was collected in EDTA-anticoagulant tubes before the morning dose of treatment and centrifuged, within the first 2 h post-extraction, at 3000 rpm for 10 min, and the plasma was stored at -70°C for batch analysis. CXCL-10 concentrations were measured by ELISA (Quantikine ELISA human CXCL-10/IP10 R&D Systems Id. DIP100, Minneapolis, MN, USA) according to the manufacturer’s instructions. The minimum detectable plasma CXCL-10 concentration was 1.67pg/mL.

### 2.6 Plasmatic miRNA analysis

At the time of the clinical visits and pharmacokinetic profiles and biopsies, plasma miR-155-5p, miR-122-5p and miR-181a-5p expression was assessed by quantitative real-time PCR (qPCR) using a LightCycler 480 Real-Time PCR System (Roche, Basel, Switzerland). Blood samples (3 ml) were collected into EDTA-K3 tubes at the pretransplantation visit and at each visit after LT according to the study design. Blood samples were obtained prior to the immunosuppressant administration (predose); at those points concurrent with rejection episodes, the samples were collected before any treatment change was made. After centrifugation (within 2 hours) at 3,000 rpm for 10 min, plasma was collected and stored in RNase-free tubes at -70°C for batched analysis.

Plasmatic expression was analyzed as previously described by our group (22). Briefly, total RNA was purified from patient plasma according to the manufacturer’s instructions (miRCURY™ RNA Isolation Kits – Biofluids from Qiagen, Hilden Germany) and reverse transcribed into cDNA. qPCR was performed using a miRCURY LNA SYBR Green PCR Kit Qiagen ID: 339347, Polyadenylation and cDNA Synthesis System (Qiagen, Hilden Germany). The amplification curves were analyzed using Roche LC Software for determining Cq by the second derivative method. ΔCq was calculated as the difference in Cq values between the miRNA target and the reference control (miR-103a-3p and miR-191-5p), following the manufacturer’s instructions; relative expression levels of target miRNAs were then evaluated within a sample according to the formula 2^(-ΔCq), where high values corresponded to higher expression.

Therapeutic drug monitoring of TAC and EVR and the analysis of all the biomarkers involved in this study were carried out in the Laboratory of Pharmacology and CIBERehd.

### 2.7 Statistical analyses

Statistical analysis was performed using SPSS software, version 23.0 (SPSS Inc., Chicago, IL, USA), except for the logistic regression, which was performed using Stata version 13.0 (StataCorp, College Station, Texas).

The samples were adjusted to fit a nonparametric distribution. Statistical differences between groups were assessed with the Mann-Whitney test and Kruskal-Wallis test, and correlations between miRNA expression, CXCL’s concentrations and clinical events were assessed with Spearman’s rho test. All data are presented as the median ± standard deviation (SD). A p value ≤ 0.05 was considered statistically significant.

The diagnostic and prognostic capacity of the biomarkers evaluated was studied by estimating the area under the ROC curve (AUROC) and its 95% confidence interval (95% CI). Cut-off points were established based on the Youden index optimization, defined as the Max (sensitivity + specificity -1). Based on these cut-off points, sensitivity, specificity, positive predictive value (PPV) and negative predictive value (NPV) values for each miRNA and chemokine were estimated.

A binary logistic regression model was performed using Stata version 16.0 (StataCorp, College Station, Texas). As explanatory variables, miRNA plasmatic expression, chemokine measurements and those clinical and laboratory variables that had a significant change in patients with rejection were used. TCMR was evaluated as binary data and used as response variable, with 0 indicating no event, and 1 indicating the occurrence of the event. A backwards stepwise strategy was used to find the best model for TCMR prognosis and diagnosis. The chosen model was further tested in the validation cohort.

## 3 Results

### 3.1 Rejection episodes

During the 12 month follow up after LT, 30 episodes of GD were found. Among them, 24 were TCMR episodes diagnosed in 22 patients (two of them had 2 episodes of TCMR). Regarding severity, 11 of them had moderate TCMR while 13 had mild TCMR. Most episodes (18) occurred during the first two weeks after LT. The remaining patients were diagnosed at month 1 (2), month 3 (2) and month 6 (2). All patients with TCMR episodes recovered with therapy and no graft loss due to rejection was registered. Fourteen patients (17.7%) were diagnosed with ABS during the follow-up. All of them were treated with either ERCP or percutaneous transhepatic cholangiodrainage. Regarding CMV, 41 patients (51.9%) developed detectable viral load throughout the first year of LT. Among these, 17 met the CMV infection criteria, and were treated according to the local protocol.

Regarding the IS protocol 56 individuals (70.9%) received a triple IS protocol with TAC, MMF and corticosteroids, while the remaining (23, 29,1%) received the double protocol with TAC and corticosteroids. Nineteen patients received EVR during the follow up. The main reasons for starting this therapy were: impaired renal function (12 patients), neurological side effects of TAC (6 patients) and, in one individual acute coronary syndrome coinciding with rejection. When stratifying by rejector and nonrejector status (**Table 1**), a higher proportion of patients transplanted for HCC exhibited rejection (50% vs. 22.8%, p=0.018). There was also a significantly higher proportion of patients without rejection who developed CMV infection (28.1% vs. 4.6%, p=0.03). The rest of the characteristics did not show any significant difference between groups.

### 3.2 Pharmacokinetics

Doses and trough concentration for TAC and EVR and the ratio TAC-C_0_/Doses at the 1^st^ week, on the 15^th^ day, and at the 1^st^, 2^nd^, 3^rd^, 6^th^, 9^th^ and 12^th^ months posttransplantation are summarized in **Table 3**.

**Table 3:** Pharmacokinetics parameters.

	No Rejectors	Rejectors	P value
	1 ^st^ Week	1 ^st^ Week	
TAC Dose (mg/day)	5,6±5,0	5,26±5,0	0.488
Cmin TAC (ng/mL)	4,0±4,4	7,0±4,31	0.053
TAC Ratio C0/dose (ng/mL)/(mg/day)	0,67±0,71	1,07±1,95	0.024
EVR Dose (mg/day)	Ø	Ø	Ø
Cmin EVR (ng/mL)	Ø	Ø	Ø
	No Rejectors	Rejectors	
	Day 15^th^	Day 15^th^	P value
TAC Dose (mg/day)	8,0±2,46	7,0±2,66	0.396
Cmin TAC (ng/mL)	6,6±3,87	4,75±1,77	0.008
TAC Ratio C0/dose (ng/mL)/(mg/day)	0,85±0,81	0,58±0,84	0.135
EVR Dose (mg/day)	Ø	Ø	Ø
Cmin EVR (ng/mL)	Ø	Ø	Ø
	No Rejectors	Rejectors	
	1 ^st^ Month	1 ^st^ Month	P value
TAC Dose (mg/day)	8,0±3,0	8,54±7,7	0.206
Cmin TAC (ng/mL)	8,15±3,21	7,70±2,19	0.747
TAC Ratio C0/dose (ng/mL)/(mg/day)	0,91±0,72	0,90±0,28	0.770
EVR Dose (mg/day)	8	Ø	Ø
Cmin EVR (ng/mL)	4.7	Ø	Ø
	No Rejectors	Rejectors	
	2 ^nd^Month	2 ^nd^Month	P value
TAC Dose (mg/day)	7,0±3,19	7,0±5,65	0.979
Cmin TAC (ng/mL)	7,05±4,67	5,95±2,90	0.543
TAC Ratio C0/dose (ng/mL)/(mg/day)	1,01±1,17	1,01±0,41	0.897
EVR Dose (mg/day)	8	Ø	Ø
Cmin EVR (ng/mL)	4	Ø	Ø
	No AR (133)	TCMAR (n=4)	No AR vs TCMAR
	3^rd^Month	3^rd^Month	P value
TAC Dose (mg/day)	6,5±2,98	8,0±3,51	0.635
Cmin TAC (ng/mL)	7,62±3,78	5,50±3,75	0.610
TAC Ratio C0/dose (ng/mL)/(mg/day)	1,02±0,98	0,97±0,47	0.513
EVR Dose (mg/day)	2±2,17	Ø	Ø
Cmin EVR (ng/mL)	4,45±2,30	Ø	Ø
	No Rejectors	Rejectors	
	6^th^Month	6^th^Month	P value
TAC Dose (mg/day)	6,0±3,21	4,50±0,71	0.470
Cmin TAC (ng/mL)	7,30±3,76	5,40±0,57	0.178
TAC Ratio C0/dose (ng/mL)/(mg/day)	1,27±1,14	1,23±0,32	0.981
EVR Dose (mg/day)	2±1,92	2.5	0.977
Cmin EVR (ng/mL)	3,8±2,06	4	0.855
	No Rejectors	Rejectors	
	9^th^Month	9^th^Month	P value
TAC Dose (mg/day)	5,0±2,68	Ø	Ø
Cmin TAC (ng/mL)	6,60±2,87	Ø	Ø
TAC Ratio C0/dose (ng/mL)/(mg/day)	1,35±0,95	Ø	Ø
EVR Dose (mg/day)	3,0±1,72	Ø	Ø
Cmin EVR (ng/mL)	4,8±2,33	Ø	Ø
	No Rejectors	Rejectors	
	12^th^Month	12^th^Month	P value
TAC Dose (mg/day)	4,5±2,86	Ø	Ø
Cmin TAC (ng/mL)	7,20±2,78	Ø	Ø
TAC Ratio C0/dose (ng/mL)/(mg/day)	1,54±1,38	Ø	Ø
EVR Dose (mg/day)	3±1,75	Ø	Ø
Cmin EVR (ng/mL)	5,05±2,05	Ø	Ø

### 3.3 Plasmatic miRNA expression of miR-155-5p, miR-181a-5p and miR-122-5p

**Figure 1:**
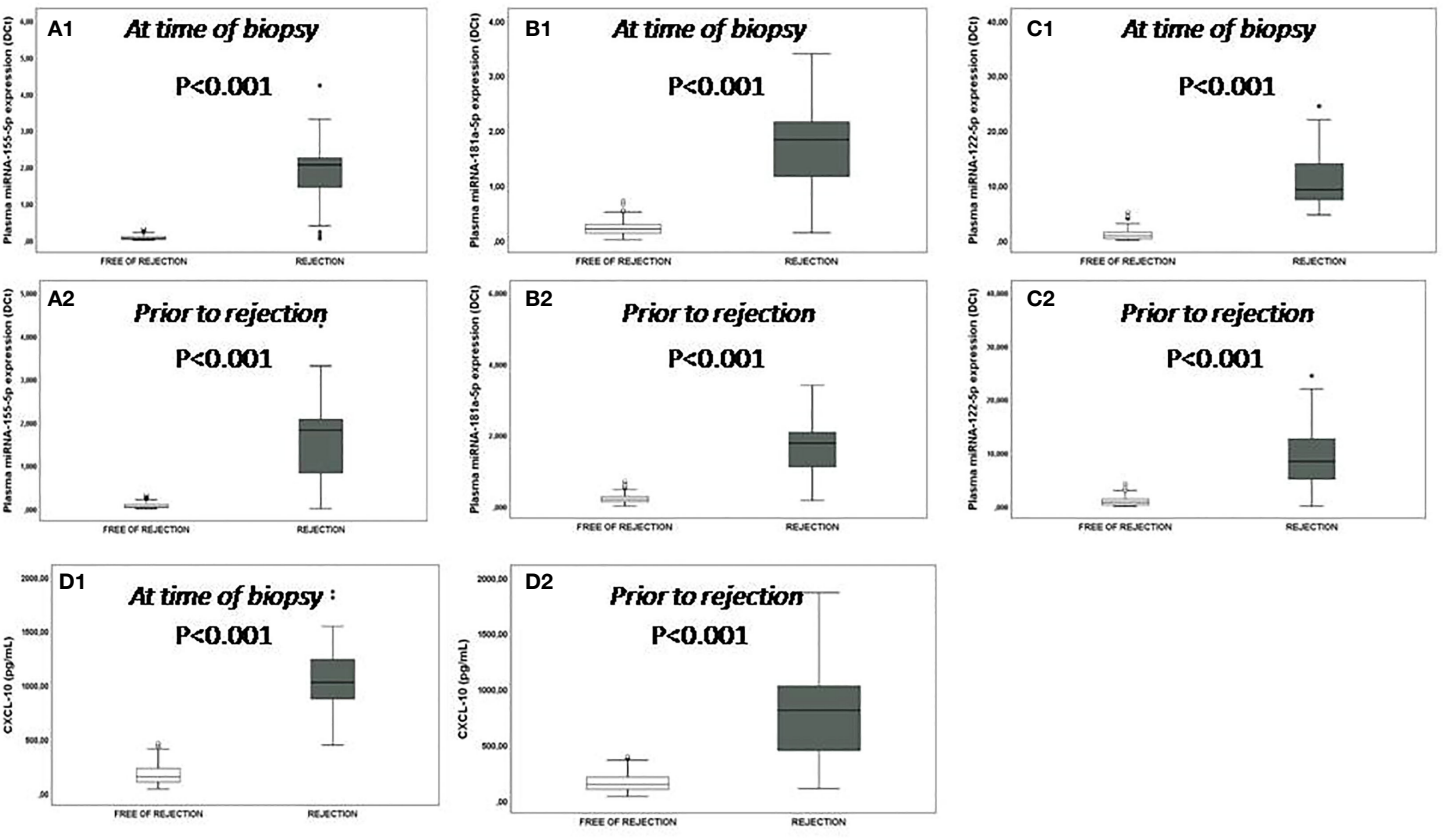
Plasmatic miRNA expression and CXCL-10 production. Plasmatic miRNA expression and CXCL-10 production at time of the biopsy: **(A1-C1)** box plots show plasma levels of miR-155-5p, miR-181a-5p and miR-122-5p between patients with rejection (n=22) (black boxes) and those without rejection (n=57) (white boxes); **(D1)** box plots show plasmatic CXCL-10 production between patients with rejection (n=22) (black boxes) and those without rejection (n=57) (white boxes). **(A2-C2)** box plots show plasma levels of miR-155-5p, miR-181a-5p and miR-122-5p and **(D2)** CXCL-10 production between patients with rejection (n=22) (black boxes) and those without rejection (n=57) (white boxes) prior to the rejection episode. The expression of miRNAs or CXCL-10 production in those patients free of rejections correspond to a post-transplant time similar to when the patients were biopsied or prior to rejection. Significant differences between groups were assessed with the Mann-Whitney test.

In the cohort of the present study, the results showed a significant increase in plasmatic expression of miR-155-5p, miR-181a-5p and miR-122-5p (p<0.001) in those patients with rejection (**Figure 1**). These significant differences in both groups not only occurred at the time of performing the biopsy (**Figures 1A1–C1**) but also beforehand (**Figures 1A2–C2**) revealing not only the diagnostic capacity of these biomarkers but also the predictive.

**Figure 2:**
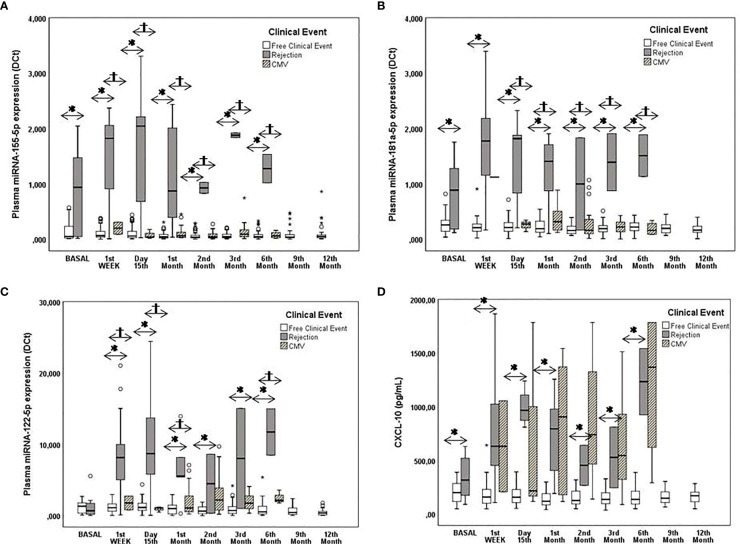
Monitoring of pre- and posttransplantation plasmatic miRNA expression and CXCL-10 production. Pre and posttransplantation plasmatic miRNA expression and CXCL-10 production. **(A–C)** Box plots show plasma levels of miR-155-5p, miR-181a-5p and miR-122-5p between patients with rejection (n=22) (black boxes) and those with CMV^+^ [replication (n=24) and infection (n=17)] (striped boxes) without clinical events (n=57) (white boxes) and **(D)** Box plots show plasma levels of CXCL-10. Significant differences between groups were assessed with the Mann-Whitney test. A value of p<0.05 was considered to indicate statistical significance: *patients free of clinical events vs. patients with rejection; ^†^patients free of clinical events vs. CMV^+^ patients.

Significant differences were observed in the pretransplant plasmatic expression of miR-155-5p (p=0.031, a median value 16.75-fold increased) and miR-181a-5p (p=0.002, a median value 3.8-fold increased) between patients with and without rejection (**Figure 2**). A significant increase in the expression of miR-155-5p, miR-122-5p and miR-181a-5p was also observed posttransplantation at the 1^st^ week, on the 15^th^ day, and at the 1^st^, 2^nd^, 3^rd^ and 6^th^ months in patients with rejection compared with patients without rejection (**Figures 2A–C**) (**Table 4**).

**Table 4:** miRs and CXCL-10 in patients with and without rejection.

	No Rejection	Rejection	P value	No Rejection	Rejection	P value	No Rejection	Rejection	P value	No Rejection	Rejection	P value	No Rejection	Rejection	P value
	Pre-Transplantation			1^st^ Week			15^th^ Day			1^st^ Month			2^nd^ Month		
**miR-155-5p**	0,056±0,27	0,937±0,78	**0.031**	0,081±0,18	1,821±0,53	**0.000**	0,059±0,20	2,043±0,98	**0.000**	0,041±0,06	0,871±0,13	**0.002**	0,036±0,07	0,927±0,14	**0.002**
**miR-181a-5p**	0,240±0,15	0,926±0,47	**0.002**	0,219±0,36	1,773±0,58	**0.000**	0,211±0,18	1,816±071	**0.000**	0,190±0,15	1,406±0,58	**0.000**	0,159±0,09	1,248±072	**0.049**
**miR-122-5p**	0,929±0,85	0,946±0,37	**0.863**	1,272±0,29	8,140±0,91	**0.000**	1,172±0,26	8,699±0,77	**0.000**	1,049±0,76	5,560±0,95	**0.011**	0,643±0,08	4,489±0,84	**0.002**
**CXCL-10**	200,31±106,46	316,75±97,33	**0.048**	159,11±106,61	631,76±107,16	**0.000**	157,44±89,57	920,48±211,37	**0.000**	119,87±80,61	793,69±129,01	**0.000**	127,05±79,67	454,076±163,66	**0.037**
	No Rejection	Rejection		No Rejection	Rejection		No Rejection	Rejection		No Rejection	Rejection				
	3^rd^ Month		P value	6^th^ Month		P value	9^th^ Month		P value	12th Month		P value			
**miR-155-5p**	0,045±0,05	1,920±0,09	**0.000**	0,470±0,76	1,275±0,36	**0.001**	0,037±0,11	Ø		0,052±0,02	Ø				
**miR-181a-5p**	0,189±0,94	1,907±0,68	**0.000**	0,219±0,09	1,509±0,53	**0.001**	0,184±0,10	Ø		0,160±0,08	Ø				
**miR-122-5p**	0,743±0,38	7,362±0,71	**0.023**	0,601±0,95	11,733±2,59	**0.001**	0,449±0,06	Ø		0,404±0,04	Ø				
**CXCL-10**	136,65±74,63	810,33±153,32	**0.002**	136,88±74,96	1232,94±236,74	**0.002**	147,86±62,6	Ø		170,76±58,38	Ø				

All data are presented as the median ± standard deviation (SD). A value of p<0.05 was considered statistically significant.

The optimal cut-off value, AUC, sensitivity, specificity, PPV and NPV are summarized in **Table 1S**. Even more, the monitoring of the individual evolution in the expression of miR-155-5p, miR-181a-5p and miR-122-5p in each patient with rejection prior to, during and after the rejection episodes showed that the expression of each miRNA progressively increased preceding the rejection episode and reached maximum levels at the time of the episode. Once the rejection episode was resolved the miRNA levels decreased (**Figure 1S**). Four patients with rejection also suffered CMV infection but in all cases, it was after the rejection episode and only a new elevation was observed for the plasmatic miR-122-5p expression, but it was much lower than that observed when there was rejection.

We attempted to determine whether CMV infection is a confounding factor for the clinical utility of plasmatic miR expression as a prognostic and diagnostic biomarker for rejection. The results showed that only miR-122-5p was 3.5-fold higher in patients with active CMV replication than in patients free of infection (**Figure 2C**).

Compared to patients free of clinical events, in patients who presented ABS, the expression of miRNA was not significantly modified. The plasmatic expression of the miRNAs was also not affected in those patients in whom EVR was introduced in the IS therapy (Data not shown).

### 3.4 Plasmatic CXCL-10 production

In the cohort of the present study a significant increase in plasmatic CXCL-10 production (p<0.001 a median value 5.6-fold increased) was observed in the rejector group (**Figure 2D**). These significant differences also occurred at the time of biopsy (**Figure D1**) and previously (**Figure D2**).

There were significant pretransplantation differences in the plasmatic CXCL-10 concentration between patients without and with rejection (p=0.048 a median value 1.5-fold increased). After transplantation, patients with rejection had significantly higher plasmatic CXCL-10 levels than those without rejection throughout the study period (p<0.05) (**Figure 2D**) (**Table 4**).

Pre and posttransplantation cut-off values for predicting rejection were determined based on AUC analysis of the ROC curve. The cut-off values obtained in the present cohort confirm those obtained in the previous cohort study (**Table 1S**).

We sought to determine whether CMV infection was a confounding factor for the clinical utility of the plasmatic CXCL-10 concentration as a prognostic and diagnostic biomarker for rejection. High interpatient variability was observed in the CMV group. Patients with CMV infection had significantly higher plasmatic CXCL-10 concentrations than CMV-free patients (p<0.01) (**Figure 2D**), reaching similar levels to those observed in patients with rejection.

No significant differences in CXCL-10 production were found in patients in whom EVR was introduced as an immunosuppressant, or in those who developed ABS.

Similar to the plasmatic expression of miRNAS, the results of an analysis of the individual evolution of plasmatic CXCL-10 concentration in each patient with rejection prior to, during and after the rejection episodes showed that this biomarker progressively increased preceding the rejection episode and reached maximum levels at the time of the episode. Once the rejection episode was resolved the CXCL-10 concentrations decreased (**Figure 2S**). Four patient with rejection also suffered CMV infection but in all cases, it was after the rejection episode and a new elevation of the plasmatic CXCL-10 concentrations was observed.

### 3.5 Development and validation of a prognostic algorithm for the risk of rejection based on miRNA-155-5p, miRNA-181a-5p and CXCL-10

#### 3.5.1 Prediction and diagnosis of TCMR

From the data obtained in the cohort of the present study a model was further developed for the prediction and diagnosis of TCMR. Clinical and laboratory parameters that had a significant change in patients with rejection were used: HCC diagnosis before LT, AST, ALT, gamma-glutamyl transpeptidase (GGT) and bilirubin. In addition, miR-155-5p, miR-122-5p, miR-181a-5p and CXCL-10 were used as explanatory variables. The final model included CXCL-10 and miR-155-5p and miR-181a-5p, by means of the following algorithm: -5.3 + 1.25 miR-155-5p + 3 miR-181a-5p + 0.001 CXCL-10. The algorithm performance for TCMR prediction and diagnosis is summarized in **Table 5**. The area under the ROC curve (AUROC) for TCMR prediction was 0.975 (95% CI 0.96;0.99) for a cut-off value of 0.4, with 79.6% sensitivity, 99.1% specificity, 90.7% PPV, 97.7 NPV and 97.1% of correctly classified individuals. For TCMR diagnosis, the AUROC was 0.99 (95% CI 0.98;0.99) for a cut-off value of 0.85, with 87.5% sensitivity, 99.5% specificity, 91.3% PPV, 99.3% NPV and 98.9% of correctly classified individuals. Regarding the correlation with the liver serum markers, we did not find a correlation when using linear regression models (neither with the algorithm, nor with each biomarker), obtaining R-squared measures below 0.1 in all cases. Given the significantly higher concentration of CXCL-10 in those patients with detectable CMV viral load in plasma, we determined that the model was significantly higher in those patients without rejection with CMV infection (0.012 vs 0.047, p<0.001). Nevertheless, in patients with rejection, the model was significantly higher than in these patients without rejection with a CMV detectable load in plasma (0.047 vs. 0.982, p<0.001). No differences were found in those patients with ABS (0.013 vs. 0.012, p=NS).

**Table 5:** Score.

	Cutoff value	AUROC (95%CI)	Se (%)	Sp (%)	PPV (%)	NPV (%)	Correcly classified (%)
Study cohort (2019)							
TCMR prediction	0.40	0.975 (0.96;0.99)	79.6	99.1	90.7	97.7	97.1
TCMR diagnosis	0.85	0.99 (0.98;0.99)	87.5	99.5	91.3	99.3	98.9
Validation cohort (2014)							
TCMR prediction	0.40	0.89 (0.82;0.96)	62.2	97	62.2	97	94.4
TCMR diagnosis	0.85	0.92 (0.89;0.94)	64.3	99.3	75	98.9	98.2

Performance of the algorithm -5.3 + 1.26 miR-155-5p + 3 miR-181a-5p + 0.001 CXCL-10 for prediction and diagnosis of TCMR. AUROC, area under the ROC curve; Se, sensitivity; Sp, specificity; PPV, positive predictive value; NPV, negative predictive value.

#### 3.5.2 Validation in an historical cohort

The algorithm was tested in the previous cohort of patients in which chemokine and miRNA analyses were available (22), comprising of 86 individuals (the characteristics of this validation cohort are shown in **Table 2**, comparing them to those of the cohort of the present study). The only features that had a significant difference were a higher proportion of patients with HCV as the primary disease (33.7% vs. 17.7%, p=0.02) and a longer ischemia time (435 vs. 393 minutes, p=0.004). The performance for prediction and diagnosis of the algorithm in the validation cohort is also summarized in **Table 5**. The AUROC for TCMR prediction was 0.89 (95% CI 0.82;0.96), with 62.2% sensitivity, 97% specificity, 62.2% PPV, 97% NPV and 94.4% of correctly classified individuals. For TCMR diagnosis, the AUROC was 0.92 (95% CI 0.89; 0.94), with 64.3% sensitivity, 99.3% specificity, 75% PPV, 98.9% NPV and 98.2% correctly classified individuals.

#### 3.5.3 Assessment of the biomarker algorithm in patients with GD submitted to a LB

The biomarker algorithm was subsequently tested in the specific setting of patients from both cohorts who were submitted to a LB due to GD with TCMR suspicion. Only patients in whom all miRNAs and CXCL-10 were available were considered for the analysis. In total, 54 episodes of GD were found, in 37 of them the TCMR diagnosis was confirmed by biopsy lecture. Among the remaining 17 episodes, the most frequent causes of GD were HCV recurrence, unspecific inflammation not meeting rejection criteria and ischemia-reperfusion injury. No significant differences were found between groups in the liver function tests (LFTs), but TAC trough levels were significantly lower in the patients with rejection (5.38 vs. 7.96 ng/ml, p=0.006) only at the 2^nd^ week after transplantation. For a cut-off value of 0.52, the AUROC for rejection diagnosis was 0.98 (95% CI 0.91;0.99), with 97.3% sensitivity, 94.1% specificity, 97.3% PPV, 94.1% NPV and 96.3% correctly classified individuals. The AUROC of the algorithm was significantly better than that of the TAC trough levels and the AUROC of ALT levels (as it showed the best diagnostic performance among the usual LFTs) (**Figure 3S**).

## 4 Discussion

In this study, we report the development of the first noninvasive score, which includes the expression of miR-155-5p, miR-181a-5p and the production of CXCL-10, for predicting and diagnosing TCMR and GD in LT patients which correctly classified 97.1% and 98.9% of the patients respectively. Such findings suggest that sequential monitoring of this plasmatic score after transplantation could reveal useful predictive and diagnostic biomarkers for TCMR in adult liver transplant recipients and differentiate TCMR from other causes of GD (CMV infection and ABS). In addition, it may avoid the need for LB, it may provide a decrease in the number of biopsies to be performed, and could help in a more efficient guide for immunosuppressive therapy adjustment.

Post transplantation, our results showed a significant increase in the expression of miR-155-5p, miR-181a-5p and miR-122-5p in patients who experienced TCMR. The AUC (>0.890), PPV (>86%) and NPV (100%) values for these miRNAs were outstanding. Thus, confirming our previous results (22) in an independent cohort regarding the clinical usefulness of monitoring plasmatic expression of miR-155-5p, miR-181a-5p and miR-122-5p as predictive and diagnostic noninvasive biomarkers for the risk of acute rejection.

In addition, patients with rejection also had significantly higher plasmatic CXCL-10 levels than those without rejection throughout the study period (AUC=0.962; %PPV: 86.7 and %NPV: 99.7), indicating an outstanding discriminatory ability of these noninvasive biomarkers to identify patients at high risk of developing TCMR. To the best of our knowledge, this study is the first in LT that has validated the capacity of plasmatic detection of CXCL-10 for the assessment of the risk of TCMR.

Focusing on the biomarkers involved in the score, miR-155 expression varies in different cell types and tissue environments and is regulated by several pathways in response to cellular signals. Previous reports have demonstrated the significantly increased expression of miR-155-5p in a variety of activated B cells, T cells and macrophages indicating its decisive role as a regulator of inflammation, immunity and tumorigenesis (36, 37). Notably, miR-155-5p regulates the imbalance of hepatic immune homeostasis, including graft rejection, viral infection, autoimmune hepatitis and septic liver injury (38). Our group (22) and others (20) have shown the potential role of miR-155-5p as a noninvasive prognostic and diagnostic biomarker of rejection (TCMR) and subclinical rejection (SCR). In addition, T-cell receptor (TCR) sensitivity and signaling strength can be modulated at the posttranscriptional level by miR-181a (39). Allospecific T-cells become activated through the interaction of their TCRs with an intact allogeneic major histocompatibility complex; the modulation of selection also argues that this miRNA might directly impact the mature T cell repertoire, which might further affect the onset and/or progression of the T-cell alloresponse. Therefore, changes in its expression can regulate and modulate the alloresponse against the implanted graft and, consequently, may play a role in the development of rejection (39, 40). Moreover it has been described that miR-181 family regulates T and B- cell development. In the present study, ours miR-181a-5p results are in agreement with those obtained in our previous cohort study and with those of other groups (41). Finally, CXCL-10 is a potent chemoattractant for several immune cells, including CD4 and CD8 T cells, to the sites of inflammation, and participates in the recruitment of alloantigen primed T cells and during the induction of proinflammatory cytokines (42). It induces, maintains, and amplifies both inflammatory and immune responses and plays a critical role in rejection (34, 43). Furthermore, all urinary noninvasive scores for the diagnostic of renal allograft rejection described thus far included CXCL-10 (8–11). Our results with respect to the upregulation of this chemokine in patients with rejection, are in agreement with those obtained in kidney transplant recipients, identifying it as a potential predictive and diagnostic biomarker for TCMR (28, 30).

Clinical confounding factors could restrict the use of these molecular biomarkers to identify those patients at risk of rejection. A significant elevation of CXCL-10 and miRNA-122-5p was found in patients with CMV infection. Thus, their role as biomarkers of rejection could lack specificity. Our results show that CXCL-10 levels were as high as those seen in patients with rejection, showing high variability concurrent with CMV replication, which could constitute a confounding factor for rejection diagnosis. When assessing the biomarker algorithm, those patients with detectable CMV viral load in plasma had significantly higher results than those without infection. This may be justified by the use of CXCL-10 as an explanatory variable in the model. However, the results in patients with rejection were even higher, allowing a clear differentiation between individuals with rejection and those without rejection with CMV infection. This provides meaning to the combination with the miRNAs that were not modified with CMV replication. In fact, it may emphasize that, as previously stated, considering that the immune response is complex and dynamic, a panel of biomarkers rather than a single biomarker might serve as noninvasive tools for rejection prediction and diagnosis. Regarding miR-122-5p, despite revealing significantly higher expression of this miR, it was not included in the biomarker algorithm. We speculate that the specificity for liver injury (44) but not particularly for rejection may justify the absence of this miR in the algorithm. On the other hand, in relation to whether the presence of ABS could be a confounding factor, the results showed that neither the miRs nor the production of CXL10 was modified by this clinical event.

It is important to remark the usefulness on monitoring the evolution in changes of these biomarkers, not only before and after TCMR was solved. In each TCMR patient, the individual expression of miR-155-5p, miR-181a-5p and miR-122-5p, as well as plasmatic CXCL-10 concentrations, gradually increased prior to the TCMR episode and reached maximum levels at the time of the episode, showing not only a diagnostic capacity of the miRNAs and CXCL-10 but also their potential utility as noninvasive predictive biomarkers.

The use of noninvasive biomarkers of rejection could be beneficial not only for predicting and diagnosing TCMR but also for optimizing of IS therapy. In the first stages after LT, careful tapering of this treatment is important for preventing both rejection and IS side effects simultaneously. In the setting of transplanted patients with normal LFTs there are no reliable parameters that may rule out the risk of rejection when reducing the IS drug doses. Prior studies in kidney transplantation showed that the monitoring of CXCL-10 could predict the development of rejection (31) showing a high NPV when the urine creatinine corrected CXCL-10 levels were low. This high NPV was also found in our work: in the present cohort and in the validation one, the NPV was 97.8% and 94.9% respectively. In this context, the biomarker model could help clinicians with safe IS drug tapering when its value is under 0.40. In contrast, as this model can be regularly monitored during the follow up of the LT patients, a rise in the values may predate rejection development and then allow a prompt IS adjustment to avoid rejection.

Another important finding of the present study was the confirmation that miRNAs (now combined with chemokines) allow the identification of patients with rejection among those with GD. After LT, particularly during the first weeks and months, it is usual to find altered LFTs in the setting of a variety of complications such as infections, biliary complications or extended ischemia reperfusion injury. Although miRNAs also showed a high predictive capacity for detecting individuals with subclinical rejection (22), given their uncertain meaning and prognosis, it is unlikely for the clinician to change IS therapy without a significant disturbance of in the LFTs. When such abnormalities become evident and there is no suspicion of other complications, the usual approach is to perform an LB to rule out rejection. Consistent with previous results (23) our findings highlight the ability of these biomarkers to discriminate patients with rejection with high accuracy (96.3% of correctly classified individuals). This capacity not only strengthens the diagnostic capacity of our model but also overcomes the lack of specificity for TCMR identification of routine LFTs and other biomarkers among patients with GD.

Regarding the robustness and feasibility of analytical methods, for the analysis of these biomarkers it is necessary to have robust, reproducible, standardized and interlaboratory validated methodologies. In the case of miRNAs and CXCL-10 both methodologies involved, RT-PCR and ELISA respectively, are feasible and easy to implement. For enzyme-linked immunosorbent assays commercial and validated kits are available and currently, most laboratories have the capacity and experience to carry out a RT-PCR. These analyses are not very labor-intensive, and the turn-around time is minimal; thus the results could be available less than 48 h. In the case of the measurement of plasmatic miRNAs RT-PCR is the hallmark method for analysis. Furthermore, the advantages of plasma markers are obvious: less invasive, involving minimal previous manipulation of the sample, samples are stable; which allows shipping between local and some international laboratories if needed and less costly in comparison with the gold standard method (biopsy).

Our study has some limitations. The event size (TCMR) was relatively small. However, the results provide confirmation on the potential role of miRNAs as predictive and diagnostic biomarkers for TCMR shown in a previous comparable demographic cohort receiving similar IS. On the other hand, we present a prospective study, with prespecified criteria for the definition of GD, in which a histological diagnosis with an expert blind revision was carried out for all patients. The study was performed in a Caucasian population, and our findings should also be validated in different ethnic populations; furthermore, the absence of patients with antibody-mediated rejection (ABMR) in our cohort did not allow us to evaluate the prognostic capacity of these miRNAs and CXCL-10 for this clinical event.

Because our analysis is only a single-center study, confirmation by other centers is needed to determine the clinical usefulness of the proposed score to predict and diagnose TCMR and differentiate from other causes of GD during the first weeks after LT.

In conclusion, the results of our study strongly suggest that the clinical implementation of this noninvasive plasmatic score, based on CXCL-10 and miR-155-5p and miR-181a-5p, may allow the prediction and diagnosis of rejection and identify patients with GD due to rejection, guiding treatment decision making in a more personalized manner and improving the quality of life of LT patients. Notably this finding warrants the development of large prospective biomarker-guided clinical trials.

## Data availability statement

The original contributions presented in the study are included in the article/**Supplementary Material**, further inquiries can be directed to the corresponding author.

## Ethics statement

The studies involving human participants were reviewed and approved by Comité de Ética de la Investigación con medicamentos (CEIm) del Hospital Clínic de Barcelona. The patients/participants provided their written informed consent to participate in this study.

## Author contributions

OM performed the miRNA and chemokine analyses, interpreted the data, performed the statistical analysis and drafted the manuscript; PR selected and followed the patients, performed the statistical analysis and drafted the manuscript; JJ performed the sample processing and storage and revised the manuscript; AL performed pharmacokinetic analysis; YF performed transplant surgeries and revised the manuscript; JC followed the patients and revised the manuscript; MN revised the manuscript; MB conceived and designed the study, interpreted the data, and drafted and revised the manuscript. All authors contributed to the article and approved the submitted version.

## Supplementary material

Supplementary Figure 1 Evolution of miR-155-5p, miR-181a-5p and miR-122-5p plasmatic expression in patients with rejection Posttransplant evolution of the miR-155-5p, miR-181a-5p and mR-122-5p plasmatic expression in patients with rejection prior to, during and after TCMR episodes. Two of them had 2 episodes of TCMR (patient #30 and #63). The solid arrow indicates the time of the TCMR episode and the dashed arrows indicate CMV replication (n=5) and CMV infection (n=1).

Supplementary Figure 2 Evolution of CXCL-10 plasma concentrations expression in patients with rejection Posttransplant evolution of CXCL-10 plasmatic concentrations in patient with rejection prior to, during and after TCMR episodes. Two of them had 2 episodes of TCMR (patient #30 and #63). The solid arrow indicates the time of the TCMR episode. The solid arrow indicates the time of the TCMR episode and the dashed arrows indicate CMV replication (n=5) and CMV infection (n=1).

Supplementary Figure 3 Comparison of the AUROC curve of the biomarker algorithm with those of TAC trough levels and ALT in t patients with GD submitted to a LBThe figure shows the comparison of the AUROCs for the diagnosis of TCMR among those patients with GD submitted to a LB. The AUROC of the biomarker algorithm including miR-155-5p, miR-181a-5p and CXCL-10 was significantly higher (p<0.001) than the AUROC of TAC trough levels and that of the ALT (which had the best performance among the usual liver function parameters).

Supplementary Table 1 ROC curve analysis: Study cohort vs. previous cohort Comparison of optimal cut-off value, AUC, 95% CI, sensitivity, specificity, PPV and NPV obtained in the cohort of the present study vs. the previous cohort.
