# Supplementary figures and images for: A plasmatic score using a miRNA signature and CXCL-10 for accurate prediction and diagnosis of liver allograft rejection
Source: Front Immunol. 2023 May 30;14:1196882. doi: 10.3389/fimmu.2023.1196882 (PMC10265684; doi:10.3389/fimmu.2023.1196882)

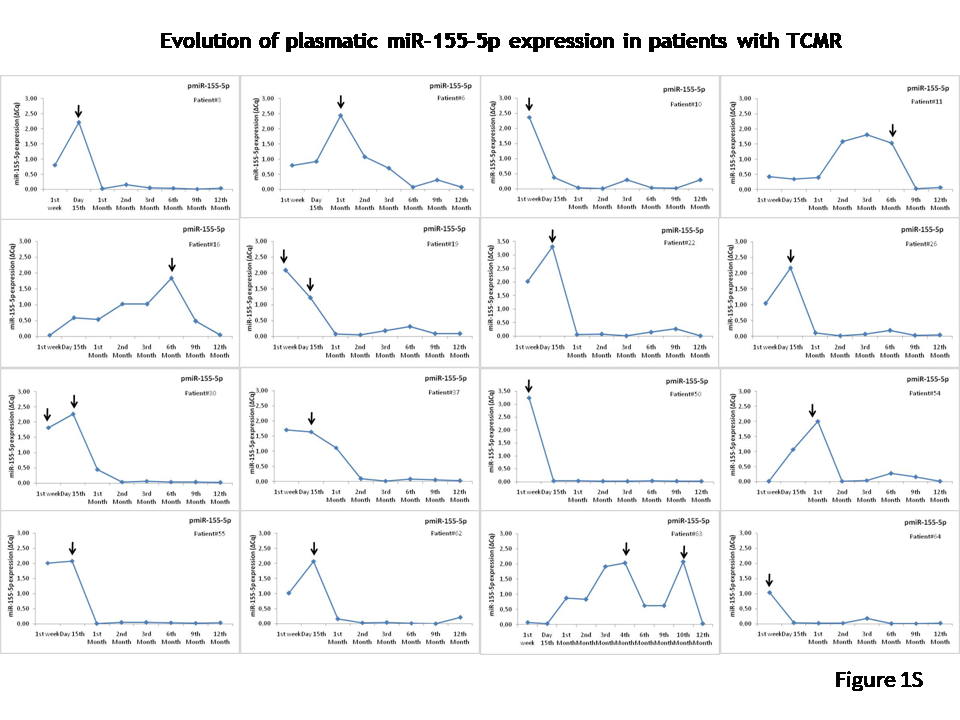

Supplement: Supplementary Figure 1 — Evolution of miR-155-5p, miR-181a-5p and miR-122-5p plasmatic expression in patients with rejection Posttransplant evolution of the miR-155-5p, miR-181a-5p and mR-122-5p plasmatic expression in patients with rejection prior to, during and after TCMR episodes. Two of them had 2 episodes of TCMR (patient #30 and #63). The solid arrow indicates the time of the TCMR episode and the dashed arrows indicate CMV replication (n=5) and CMV infection (n=1). [file Image_1.tif]

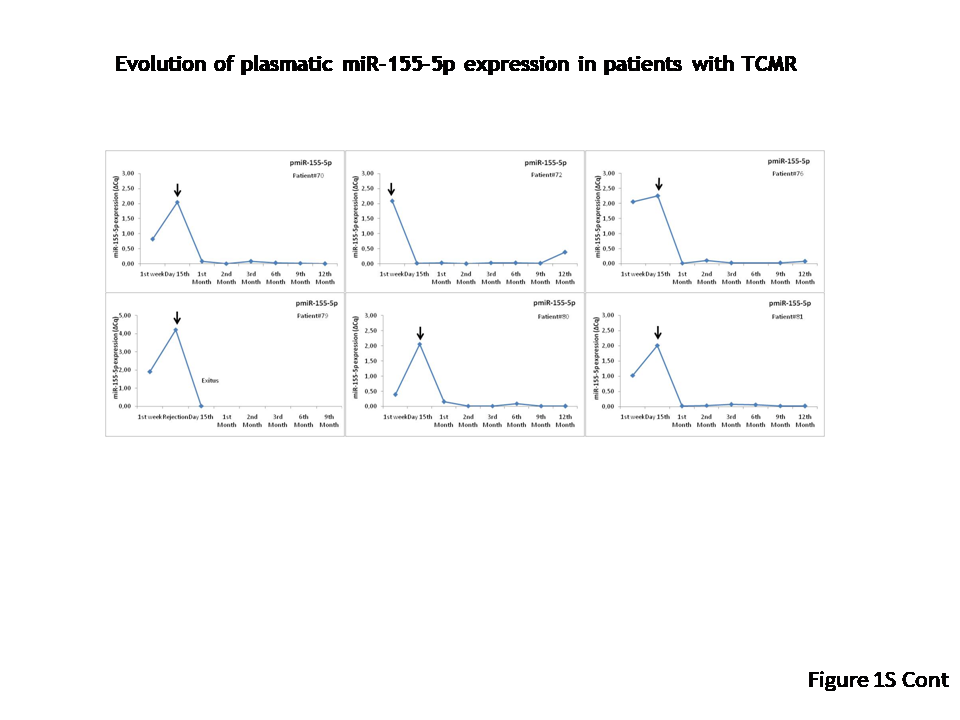

Supplement: Supplementary Figure 2 — Evolution of CXCL-10 plasma concentrations expression in patients with rejection Posttransplant evolution of CXCL-10 plasmatic concentrations in patient with rejection prior to, during and after TCMR episodes. Two of them had 2 episodes of TCMR (patient #30 and #63). The solid arrow indicates the time of the TCMR episode. The solid arrow indicates the time of the TCMR episode and the dashed arrows indicate CMV replication (n=5) and CMV infection (n=1). [file Image_2.tif]

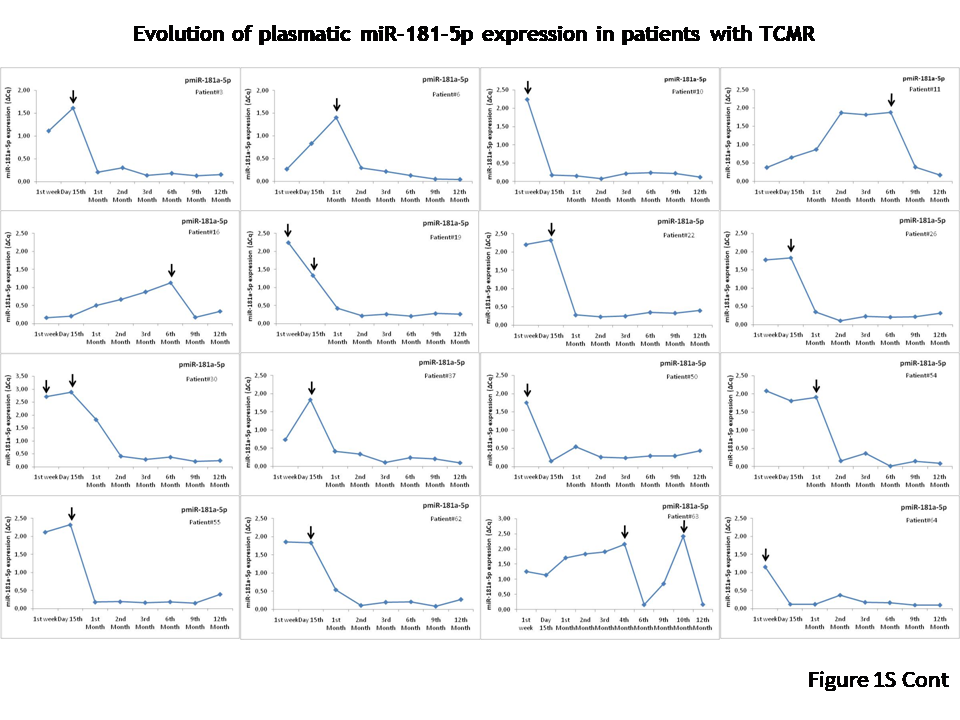

Supplement: Supplementary Figure 3 — Comparison of the AUROC curve of the biomarker algorithm with those of TAC trough levels and ALT in t patients with GD submitted to a LBThe figure shows the comparison of the AUROCs for the diagnosis of TCMR among those patients with GD submitted to a LB. The AUROC of the biomarker algorithm including miR-155-5p, miR-181a-5p and CXCL-10 was significantly higher (p<0.001) than the AUROC of TAC trough levels and that of the ALT (which had the best performance among the usual liver function parameters). [file Image_3.tif]

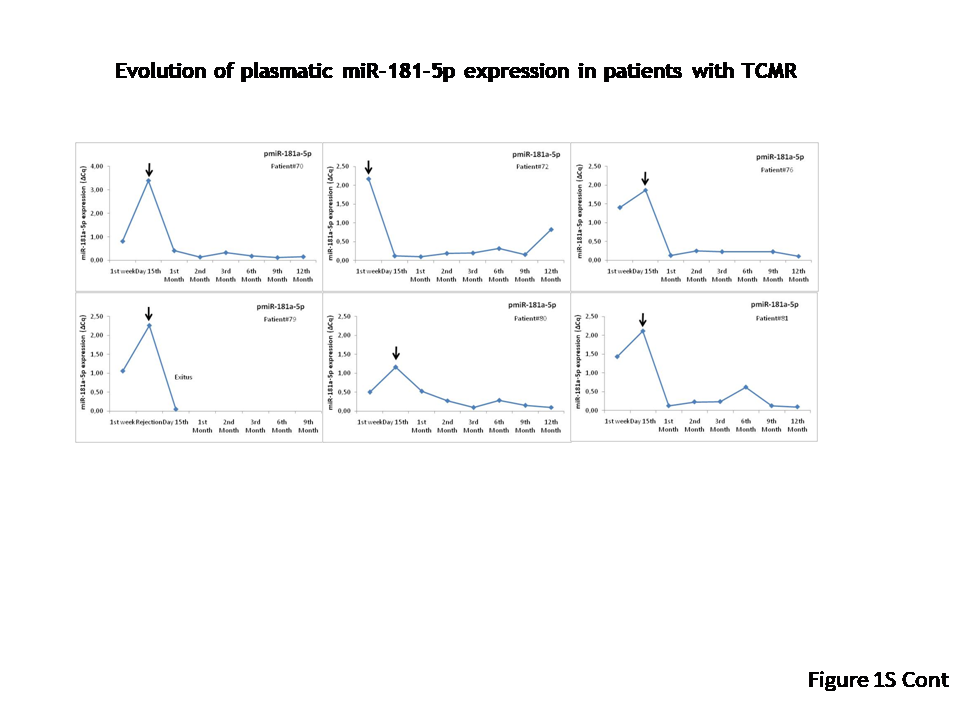

Supplement: Supplementary file 5 [file Image_4.tif]

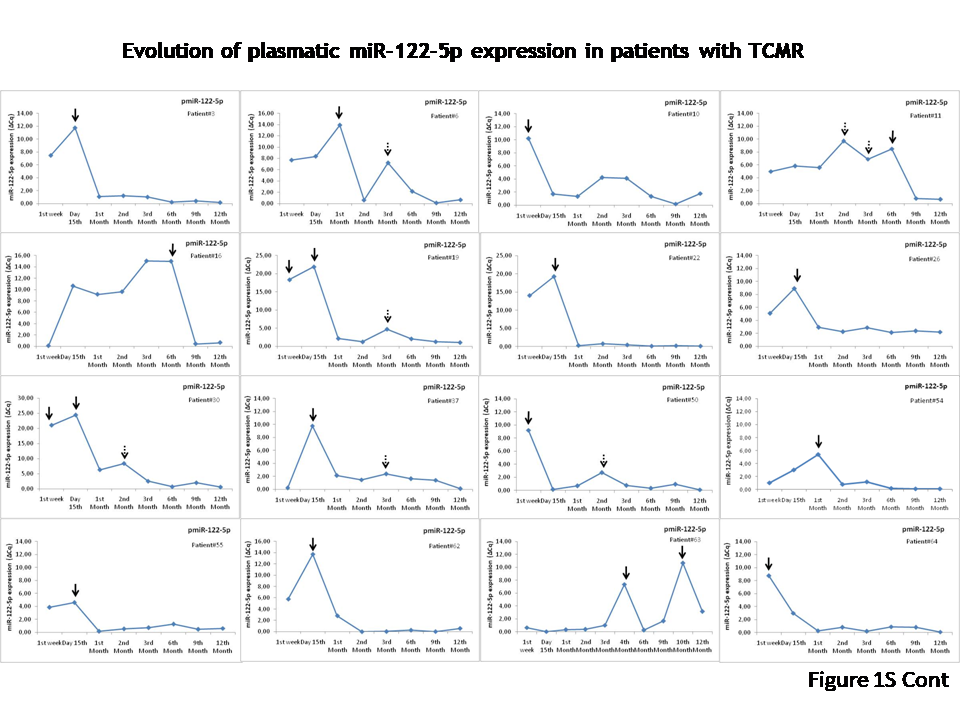

Supplement: Supplementary file 6 [file Image_5.tif]

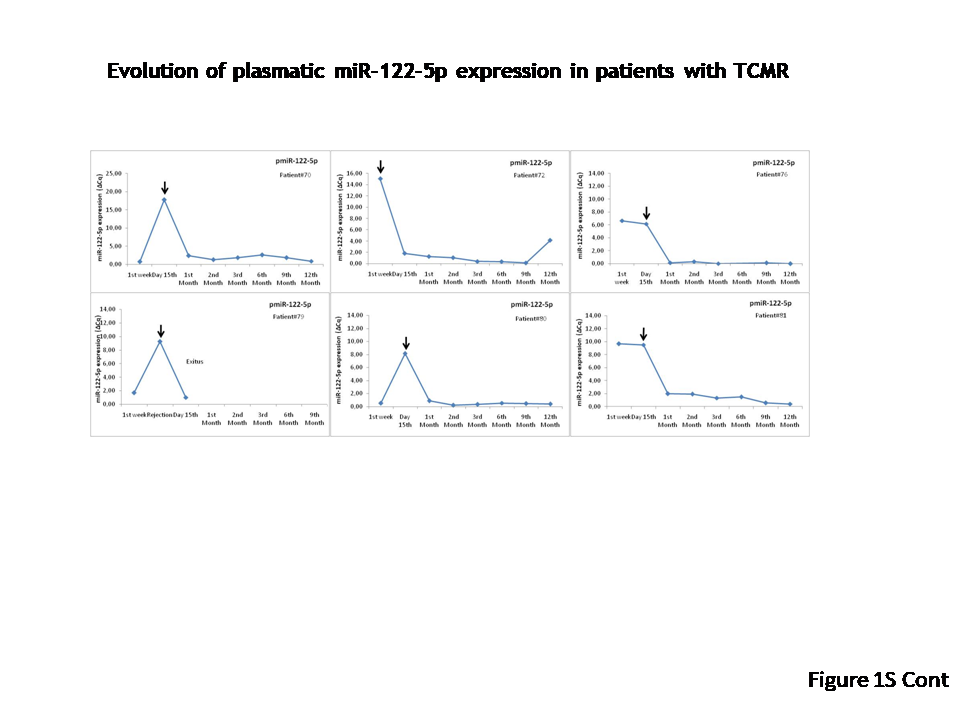

Supplement: Supplementary file 7 [file Image_6.tif]

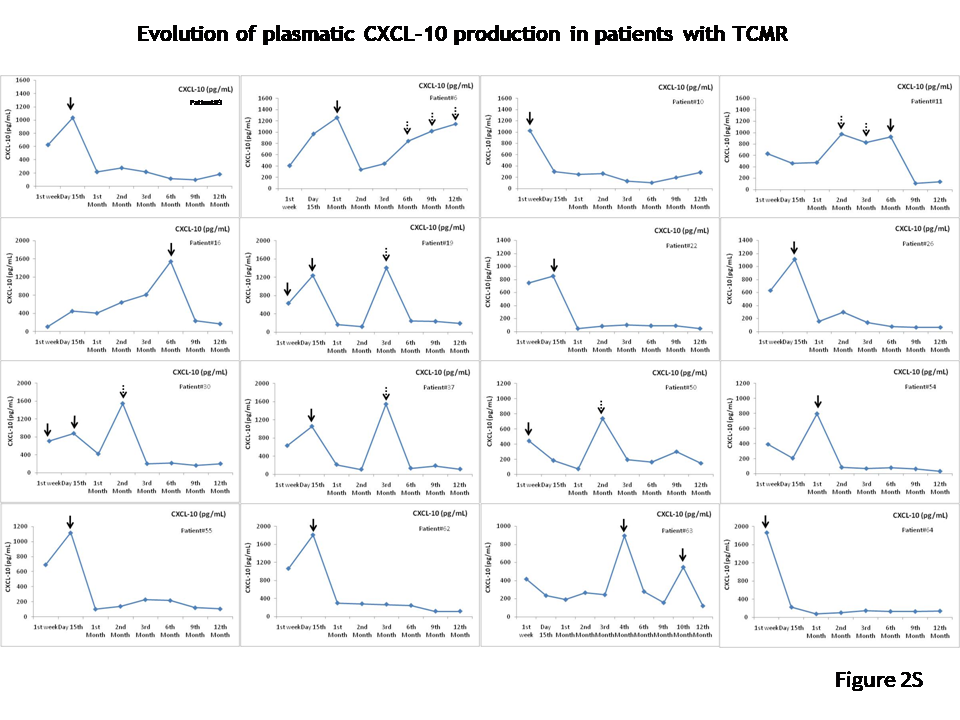

Supplement: Supplementary file 8 [file Image_7.tif]

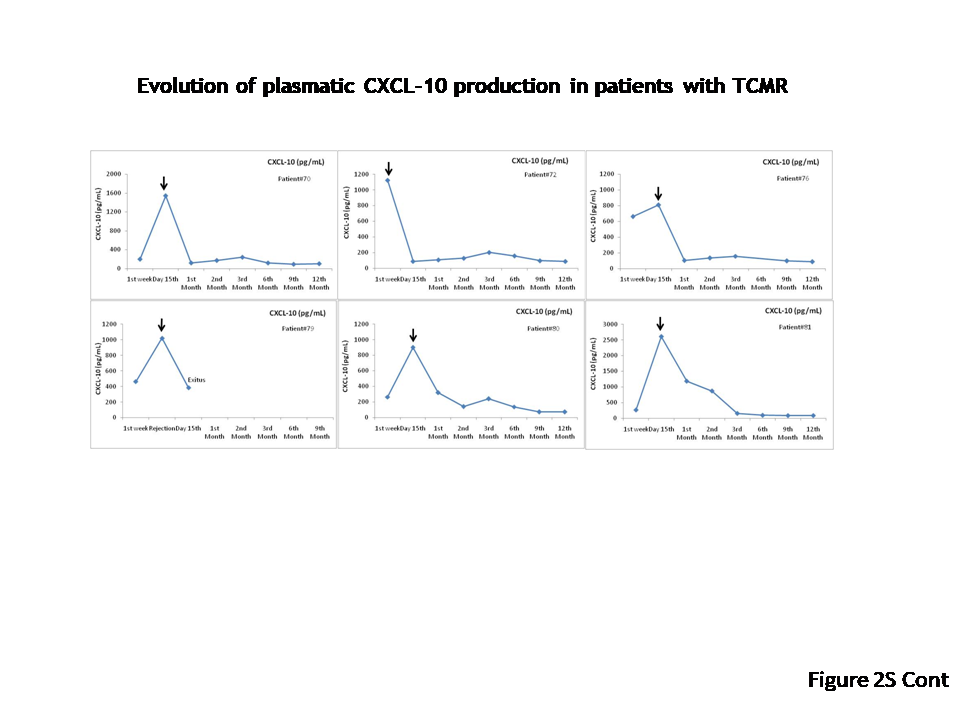

Supplement: Supplementary file 9 [file Image_8.tif]

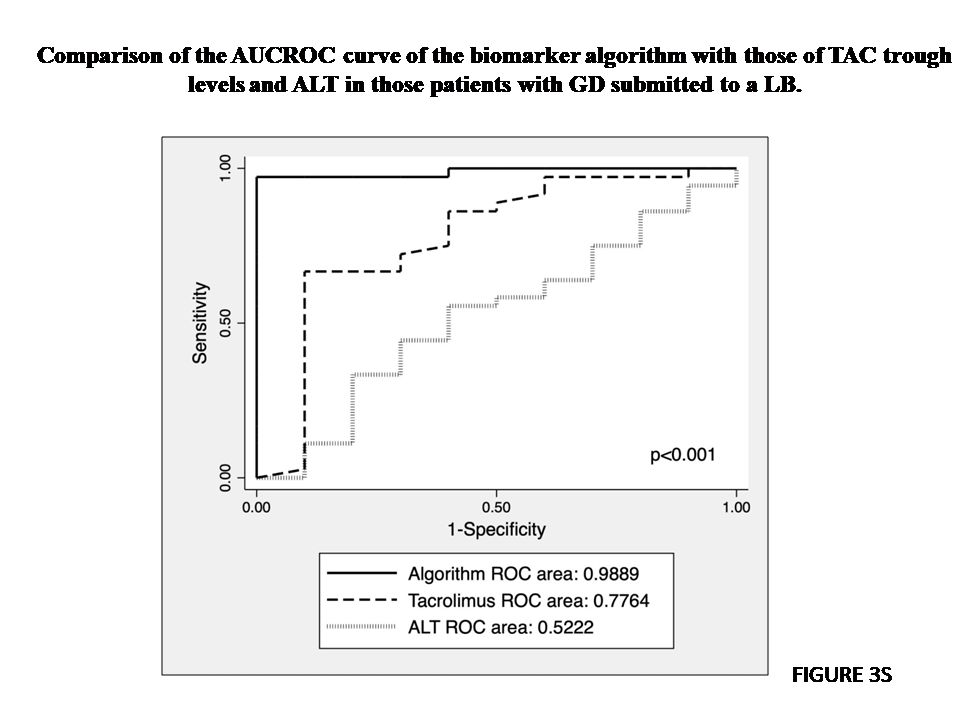

Supplement: Supplementary file 10 [file Image_9.tif]
